# Overexpression of SIRT1 and induction of apoptosis by its inhibition in adult T-cell leukemia cells

**DOI:** 10.1186/1742-4690-8-S1-A166

**Published:** 2011-06-06

**Authors:** Tomohiro Kozako, Akiyoshi Aikawa, Teruhisa Shoji, Makoto Yoshimitsu, Hiroshi Shimeno, Shinji Soeda, Kimiharu Uozumi, Naomichi Arima

**Affiliations:** 1 Department of Biochemistry, Faculty of Pharmaceutical Sciences, Fukuoka University, Fukuoka, Japan; 2 Department of Hematology and Immunology, Kagoshima University Hospital, Kagoshima, Japan; 3 Division of Hematology and Immunology, Center for Chronic Viral Diseases, Graduate School of Medical and Dental Sciences, Kagoshima University, Kagoshima, Japan

## 

Adult T-cell leukemia-lymphoma (ATL) is an aggressive peripheral T-cell neoplasm that develops after long-term infection with human T-cell leukemia virus (HTLV-1). SIRT1, a nicotinamide adenine dinucleotide (NAD+)-dependent histone/protein deacetylase, plays a crucial role in various physiological processes, such as aging, metabolism, neurogenesis and apoptosis, due to its ability to deacetylate numerous substrates, such as histone and NF-κB, which is implicated as an exacerbation factor in ATL. Here, we assessed how SIRT1 is regulated in primary ATL cells and leukemic cell lines. SIRT1 expression in ATL patients was significantly higher than that in healthy controls, especially in the acute type. Sirtinol, a SIRT1 inhibitor, induced significant growth inhibition or apoptosis in cells from ATL patients and leukemic cell lines, especially HTLV-1-related cell lines. Sirtinol-induced apoptosis was mediated by activation of the caspase family, and degradation of SIRT1 in the nucleus. Interestingly, NAD+ augmented sirtinol-induced apoptosis in leukemic cell lines. Thus, the SIRT1 inhibitor acted as a tumor suppressor, where NAD+ accelerated the SIRT1 inhibitor-induced apoptosis. These results suggest that SIRT1 is a crucial anti-apoptotic molecule in ATL cells, and that SIRT1 inhibitors may be useful therapeutic agents for leukemia, especially in patients with ATL.

